# Insights Into the Mn(VII) and Cr(VI) Adsorption Mechanisms on Purified Diatomite/MCM-41 Composite: Experimental Study and Statistical Physics Analysis

**DOI:** 10.3389/fchem.2021.814431

**Published:** 2022-02-08

**Authors:** Inas A. Ahmed, Michael Badawi, Adrián Bonilla-Petriciolet, Eder C. Lima, Moaaz K. Seliem, Mohamed Mobarak

**Affiliations:** ^1^ Department of Chemistry, Faculty of Science, King Khalid University, Abha, Saudi Arabia; ^2^ Laboratoire de Physique et Chimie Théoriques, UMR 7019—CNRS, Université de Lorraine, Nancy, France; ^3^ Instituto Tecnológico de Aguascalientes, Aguascalientes, Mexico; ^4^ Postgraduate Program in Mine, Metallurgical and Materials Engineering (PPGE3M), School of Engineering, Federal University of Rio Grande do Sul (UFRGS), Porto Alegre, Brazil; ^5^ Institute of Chemistry, Federal University of Rio Grande do Sul (UFRGS), Porto Alegre, Brazil; ^6^ Faculty of Earth Science, Beni-Suef University, Beni-Suef, Egypt; ^7^ Physics Department, Faculty of Science, Beni-Suef University, Beni-Suef, Egypt

**Keywords:** purified diatom/MCM-41, manganese, hexavalent chromium, adsorption, statistical physics modeling

## Abstract

In this study, a purified diatomite (PD) with a concentration of diatom frustules more than 92% SiO_2_ was utilized to synthesize a composite of MCM-41 silica under hydrothermal conditions. The as-synthesized PD/MCM-41 composite was characterized and tested as an adsorbent for the removal of Cr(VI) and Mn(VII) ions from aqueous solution. Results of X-ray diffraction (XRD), scanning electron microscopy (SEM), transmission electron microscopy (TEM), and Fourier-transform infrared spectroscopy (FTIR) revealed that the diatom frustules of the PD were coated with MCM-41 mesoporous silica. Experimental isotherms of Cr(VI) and Mn(VII) adsorption were fitted to classical and advanced statistical physics models at 25°C–55°C and pH 3. The Langmuir model estimated monolayer adsorption capacities ranging from 144.1 to 162.2 mg/g for Cr(VI) and 166.2 to 177.0 mg/g for Mn(VII), which improved with increasing the solution temperature. Steric and energetic parameters obtained from a monolayer adsorption model with one adsorption site was utilized to explain the adsorption mechanism at a microscopic level. The number of Cr(VI) and Mn(VII) ions adsorbed on PD/MCM-41 active site (*n*) were 1.25–1.27 for Cr(VI) and 1.27–1.32 for Mn(VII), thus suggesting multi-interaction mechanisms. The density of PD/MCM-41 active sites (*D*_M_) was a key parameter to explain the adsorption of these heavy metals. The adsorbed quantities were maximum at 55°C, thus obtaining 102.8 and 110.7 mg/g for Cr(VI) and Mn(VII), respectively. Cr(VI) and Mn(VII) adsorption energies ranged from 18.48 to 26.70 kJ/mol and corresponded to an endothermic adsorption with physical forces. Entropy, free enthalpy, and internal energy associated to the adsorption of Cr(VI) and Mn(VII) ions were calculated, thus indicating that the removal of these pollutants was spontaneous. Overall, this article offers new interpretations for the Cr(VI) and Mn(VII) adsorption mechanisms on PD/MCM-41 composite, which are relevant to contribute to the development of effective water treatment processes.

## Introduction

During the last few decades, a significant amount of industrial effluents and solid has been discharged into the environment, especially the aquatic systems, due to industrialization and anthropogenic activities. Pollution of water resources *via* these effluents that contain toxic organic and inorganic compounds has generated technological challenges in terms of water treatment to reduce and minimize the associated environmental impacts and the potential harmful effects to human as well as aquatic organisms (Inamuddin and Ismail, 2010; Kabiri et al., 2014; Ahamed et al., 2016; Mohammad et al., 2016; Inamuddin et al., 2017; Fegade et al., 2020). In particular, the continuous discharge of industrial effluents that contain several heavy metal ions into water resources is a significant threat to the aquatic environment (Hua et al., 2012; Inamuddin et al., 2017). Chronic exposure to low concentrations of stable metal ions (e.g., manganese and chromium) is dangerous for human beings (Liu et al., 2019). Manganese-polluted water is associated with various products such as fireworks, batteries, alloys, glass, ceramics, and pigments (Meena et al., 2005). Drinking water containing manganese ions with a concentration higher than 0.1 mg/l can cause lung and liver diseases (Taffarel and Rubio, 2009). On the other hand, electroplating, steel fabrication leather, paints, and textile industries are considered as the main sources that can contribute to increase the concentration of chromium ions in water bodies (Selim et al., 2018). Damage of nerve tissues and cancer of the lungs and liver are also attributed to water sources with a chromate concentration higher than 0.05 mg/l (Selim et al., 2018). The development of effective procedures for the decontamination of metallic ion-bearing wastewaters and reusing them in different fields of life would be an important issue. Moreover, the utilization of accessible, eco-friendly, and low-cost raw materials as adsorbents for the removal of these metallic ions (i.e., permanganate and chromate) is recommended (Mobarak et al., 2021). Numerous studies on wastewater purification reported that adsorption is a desired technique for the removal of water contaminants in comparison to biological treatment, advanced oxidation, coagulation, irradiation, ultrafiltration, and ozonation methods (Seliem and Komarneni, 2016). Besides, the high-efficiency and simple design, wide accessibility of natural materials, and low operating cost are important factors associated with the application of adsorption strategy in water remediation (Meena et al., 2005; Seliem and Komarneni, 2016; Selim et al., 2018; Sellaoui et al., 2021a). In this direction, the modeling of the adsorption equilibrium is mandatory to characterize and understand the physicochemical factors that could govern the removal of water pollutants in adsorption systems (Li et al., 2019; Seliem and Mobarak, 2019; Mohamed et al., 2020; Ramadan et al., 2021). It is essential to explain that the analysis of isotherm parameters *via* the conventional equations (e.g., the Langmuir and Freundlich models) is not enough in the molecular-scale interpretation of the adsorption mechanism (Barakat et al., 2020; Li et al., 2020; Abu Sharib et al., 2021). For instance, the Langmuir theory establishes that the interactions between the adsorbed ions and the adsorbent active sites are homogeneous (i.e., they have the same energy) and each adsorption site can receive only one adsorbate molecule or ion (Seliem and Mobarak, 2019; Ramadan et al., 2021). The scientific meaning associated with experimental parameters such as the ion concentration and solution temperature is not clearly elucidated *via* the assumptions of these classical models (Li et al., 2020; Ramadan et al., 2021). Overall, the theory of the Langmuir and Freundlich models is inadequate to describe the interface (e.g., multi-interactions versus multi-docking) of adsorbates‒solids systems, particularly in composites (Sellaoui et al., 2018; Touihri et al., 2021). On the contrary, the fitting of experimental data to the statistical physics models (SPMs) can offer substantial theoretical factors like the number of the adsorbed ions per one functional group of the adsorbent (*n*), the density of active sites of the adsorbent (*D*_M_), the saturation adsorption capacity of the adsorbent ( $Q_{o}$ ), and the adsorption energy (Δ*E*) (Barakat et al., 2020; Li et al., 2020). Therefore, SPMs can be used to outline the interactions at the micro- and macroscopic levels, and thus, they can offer deep and novel insights into the adsorption mechanism (Li et al., 2020; Abu Sharib et al., 2021).

Diatomites are mainly composed of amorphous hydrated silica (SiO_2_ · *n*H_2_O) associated with different impurities including silica sand, clay minerals, carbonates, iron oxides, and organic matter. Thermal, chemical, or thermo-chemical techniques are used to obtain high-grade purified diatomite (PD) that contains more than 95% of SiO_2_ · *n*H_2_O (Ibrahim et al., 2013; Alyosef et al., 2014; Mohamed et al., 2019). Purified diatom is characterized by high porosity, low density, small particle size, high surface area, and chemical stability, and, consequently, it can be employed in water treatment (Ibrahim et al., 2013; Mohamed et al., 2019). A previous study showed that the thermo-chemical activation (where diatomite was treated at 900°C/3 h followed by H_2_SO_4_ interaction) of low-grade Egyptian diatomite increased the concentration of diatom frustules to 92.58% SiO_2_ and also removed the calcite (CaCO_3_) mineral (Ibrahim et al., 2013). This PD displayed an open porous structure free from CaCO_3_ impurities. This PD sample was used as an adsorbent for Cr(VI) at room temperature. However, the adsorption mechanism of Cr(VI) was only considered *via* the application of classical models (e.g., Freundlich and Langmuir) at 25°C (Mohamed et al., 2019), while the theoretical treatment was not utilized in this previous study. The main objectives of the current article were 1) to integrate the data analysis provided by traditional and advanced statistical physics models to clarify the efficiency of PD/MCM-41 composite for the adsorption of Mn(VII) and Cr(VI) at different temperatures (i.e., 25°C, 40°C, and 55°C) and 2) to interpret the steric, energetic, and thermodynamic functions (entropy, free enthalpy, and internal energy) controlling the Cr(VI) and Mn(VII) uptake mechanisms at all temperatures. Overall, the current study provides new insights and deep interpretations of the interactions between the studied metal ions and the PD/MCM-41 active sites.

## Synthesis and Characterization of the PD/MCM-41 Composite

The purified diatomite (i.e., SiO_2_ concentration >92%), cetyltrimethylammonium bromide (CTAM, Aldrich, 99%), ammonia solution, and distilled water were used as starting materials in the present study. The following method was used to prepare the PD/MCM-41 composite (Selim et al., 2018): 2.04 g of CTAM were completely dissolved in 50 ml of distilled water, and then, 32.68 ml of aqueous ammonia solution was added under continuous stirring for 30 min; 3.0 g of the purified diatomite was added to this solution with continuous stirring for 45 min. The formed mixture was hydrothermally treated at 110°C for 48 h in an electrical oven. The solid phase was separated by centrifugation, washed with distilled water, dried at 70°C, and stored for its characterization and application in adsorption studies.

X-ray diffraction (XRD) patterns of the PD/MCM-41 composite were determined using a Philips APD-3720 diffractometer. The morphological features of this composite were studied using scanning electron microscopy (SEM) and transmission electron microscopy (TEM) (JSM-6700F, JEOL, Japan). The functional groups of the PD/MCM-41 composite were identified in the range of 400–4,000 cm^−1^ *via* Fourier-transform infrared spectroscopy (Bruker FTIR-2000 Spectrometer).

## Cr(VI) and Mn(VII) Adsorption Isotherms and Modeling Analysis

Standard solutions (1,000 mg/l) of Cr(VI) and Mn(VII) were prepared and diluted by distilled water to prepare different initial adsorbate concentrations ranging from 20 to 150 mg/l. Isotherm studies of Cr(VI) and Mn(VII) were performed at these concentrations using 50 ml of metal solutions and 25 mg of PD/MCM-41 dose at pH 3 and three temperatures (25°C, 40°C, and 55°C). Selection of this pH was due to the pH of point of zero charge of the used PD/MCM-41 (pH_PZC_ = 6.97) and the dominant form of each adsorbate at the tested pH [i.e., HCrO_4_^−^ for Cr(VI) and MnO4^–^ for Mn(VII), respectively] (Selim et al., 2018; Mohamed et al., 2019). The metal‒PD/MCM-41 suspensions were mixed at 120 rpm for 2 h with a reciprocating SHO-2D rotary shaker. Both Cr(VI) and Mn(VII) adsorption quantities were calculated at equilibrium (*q*_e_, milligrams per gram) $\left(q_{e},\;\mathrm{mg}/g\right)$ via the following expression

$$q_{e}=\left(C_{0}-C_{e}\right)\frac{V}{m}$$ (1)

where *m* is the PD/MCM-41 mass (grams), *V* is the metal solution volume (liters), and $\;C_{e}$ and $C_{0}$ (milligrams per liter) are the equilibrium and initial Cr(VI) and Mn(VII) concentrations, respectively.

Langmuir (1916) and Freundlich (1906), the most common and used adsorption models, were applied to analyze the adsorption data. Cr(VI) and Mn(VII) adsorption isotherms were fitted to the non-linear forms of these models, and the values of the determination coefficient ( $R^{2}$ ) and chi-square ( $\chi^{2}$ ) were utilized to identify the best model; see Table 1.

**TABLE 1:** Classical isotherm models used to analyze Cr(VI) and Mn(VII) adsorption onto the PD/MCM-41 composite.

Isotherm model	Formula	Parameters	References
Langmuir	$q_{e}=\frac{q_{max\;K_{L}C_{e}\;}}{\left(1+K_{L}C_{e}\;\right)}$	*C*_e_ (mg/l): equilibrium concentration of Cr(VI) and Mn(VII) in the solution	Langmuir (1916)
		*q*_e_ (mg/g): adsorbed amount of Cr(VI) and Mn(VII) at equilibrium	
		*q*_max_ (mg/g): maximum adsorption capacity	
		*K*_L_ (l/mg): Langmuir constant	
Freundlich	$q_{e}=K_{F}C_{e}^{1/n}$	*K*_F_ [(mg/g)(mg/l)^–1/n^]: Cr(VI) and Mn(VII) are the Freundlich constants. *n*: heterogeneity factor	Freundlich (1906)
$R^{2}=1-\frac{\sum {\left(q_{e,\exp}-q_{e,\mathrm{cal}}\right)}^{2}}{\sum {\left(q_{e,\exp}-\;q_{e,\mathrm{mean}}\right)}^{2}}$		*q*_e, exp_ (mg/g): Experimental adsorption capacity	Ramadan et al. (2021)
$\chi^{2}=\sum \frac{{\left(q_{e,cal}-q_{e,exp}\right)}^{2}}{q_{e,exp}}$		*q*_e, cal_ (mg/g): Calculated adsorption capacity	Ramadan et al. (2021)

In this study, the experimental data of Cr(VI) and Mn(VII) were modeled *via* different SPMs to calculate theoretical parameters for the analysis of the adsorption mechanisms. These models were as follows: a monolayer model with one adsorption energy (Model 1), a monolayer model with two adsorption energies (Model 2), a double-layer model with one adsorption energy (Model 3), and a double-layer model with two adsorption energies (Model 4). The expressions used to calculate the adsorbed quantities for the four adsorption models are given in Table 2.

**TABLE 2:** Statistical physics models for the Mn(VII) and Cr(VI) adsorption on PD/MCM-41 composite.

Advanced SPMs	Formula	Parameters		Refs
Model 1	$Q=nN_{o}=\frac{nD_{M}}{1+{\left(\frac{c_{1/2}}{c}\right)}^{n}}=\frac{Q_{o}}{1+{\left(\frac{c_{1/2}}{c}\right)}^{n}}$	*Q* (mg/g): adsorbed quantity, *n*: number of ions adsorbed per adsorption site, and *D*_M_ (mg/g): receptor site density		Li et al. (2019)
		*Q*_0_ (mg/g): adsorbed quantity at saturation		
		$c_{1/2}$ (mg/l): the concentration at half-saturation		
Model 2	$Q=\frac{n_{1}D_{1M}}{1+{\left(c_{1}/c\right)}^{n_{1}}}+\;\;\frac{n_{2}D_{2M}}{1+{\left(c_{2}/c\right)}^{n_{2}}}$	*c*_1_ and *c*_2_ (mg/l): concentrations at half-saturation for the first and the second active sites, respectively		Seliem and Mobarak (2019)
		*n*_1_ and *n*_2_ (–): number of ions adsorbed per first and second adsorption sites, respectively		
Model 3	$Q=Q_{o}\frac{{\left(\frac{c}{c_{1/2}}\right)}^{n}+2{\left(\frac{c}{c_{1/2}}\right)}^{2n}}{1+{\left(\frac{c}{c_{1/2}}\right)}^{n}+{\left(\frac{c}{c_{1/2}}\right)}^{2n}}$			Li et al. (2019)
Model 4	$Q=Q_{o}\frac{{\left(\frac{c}{c_{1}}\right)}^{n}+2{\left(\frac{c}{c_{2}}\right)}^{2n}}{1+{\left(\frac{c}{c_{1}}\right)}^{n}+{\left(\frac{c}{c_{2}}\right)}^{2n}}$			Li et al. (2020)
$\mathrm{RMSE}=\sqrt{\frac{\sum_{i=1}^{m}{\left(Q_{i\;cal}-Q_{i\;exp}\right)}^{2}}{m^{'}-p}}$			$m^{\prime }:$ the experimental data number	Selim et al. (2018)
			p : the adjustable parameters	
			*Q*_i cal_ and *Q*_i exp_: the calculated and the experimental adsorbed amounts, respectively	

Concerning the number of the formed adsorbate layers (i.e., one or two), type of active sites (i.e., similar or different), and the adsorption energies (Δ*E*), the following cases can be identified:

➢ Model 1: PD/MCM-41 has one type of active site, and adsorption occurs in the form of a single layer with one energy.
➢ Model 2: PD/MCM-41 has two types of active sites, and adsorption occurs in the form of a single layer with two energies.
➢ Model 3: Mn(VII) or Cr(VI) adsorption on PD/MCM-41 is assumed to occur forming two layers with the same adsorption energy (Δ*E*_1_).
➢ Model 4: The adsorption is theorized to occur *via* the formation of two Mn(VII) or Cr(VI) layers with two energies (Δ*E*_1_ and Δ*E*_2_). ΔE_1_ describes the PD/MCM-41–metal interaction, and it is higher than ΔE_2_, which signifies the metal–metal interface.

The suitability of these advanced SPMs to fit the Cr(VI) and Mn(VII) experimental data was compared, and the best monolayer or double-layer model was selected via the $R^{2}$ and root mean square error values RMSE (RMSE); see Table 2 (Selim et al., 2018; Mobarak et al., 2019; Barakat et al., 2020; Mohamed et al., 2020; Abu Sharib et al., 2021).

## Results and Discussions

### Characterization of PD/MCM-41 Composite

**FIGURE 1:**
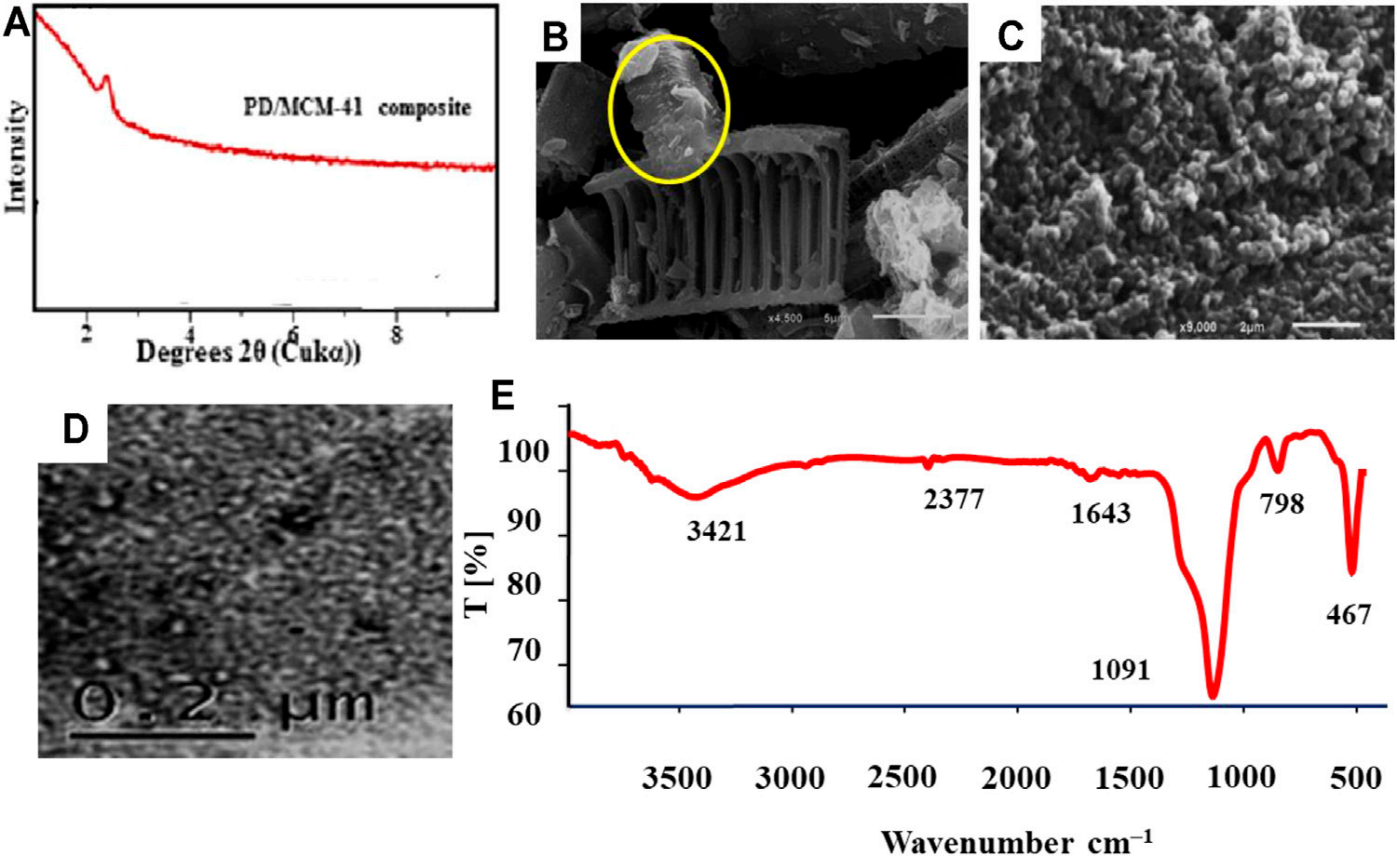
Characterization of the PD/MCM-41 silica composite: **(A)** X-ray diffraction (XRD) patterns, **(B, C)** scanning electron microscopy (SEM) and **(D)** transmission electron microscopy (TEM) photographs, and **(E)** Fourier-transform infrared spectroscopy (FTIR) spectrum.

Characterization of the composite (PD/MCM-41) *via* XRD, SEM, TEM, and Fourier-transform infrared spectroscopy (FTIR) techniques was considered in a previous study (Selim et al., 2018). Overall, the results of XRD, SEM, and TEM are displayed in Figure 1. The main peak (100) observed at low angle (2*θ* of 2.4°) supported the preparation of MCM-41 silica (Figure 1A). SEM indicated that the diatom frustules of the PD were coated by particles of MCM-41 mesoporous silica, thus having different shapes and sizes (Figure 1B, C). The hexagonal mesostructure of MCM-41 silica and its composite with purified diatomite is presented by a TEM image (Figure 1D). FTIR spectrum of the PD/MCM-41 showed different absorption bands at 3,421, 2,923, 2,853, 2,377, 1,643, 1,091, 798, and 467 cm^−1^ (Figure 1E). The –OH group was detected at 3,421 cm^−1^, while the stretching C–H groups (symmetric and asymmetric) could be associated to the bands at 2,853 and 2,923 cm^−1^. The weak band observed at 1,643 cm^−1^ suggested the existence of H–O–H bending vibration of water molecules. The very strong band located at 1,091 cm^−1^ could be related to the Si–O–Si stretching group. Also, the observed strong bands at 798 and 456 cm^−1^ could be associated to the Si–O–Si siloxane groups.

### Modeling of Cr(VI) and Mn(VII) With Langmuir and Freundlich Equations

**FIGURE 2:**
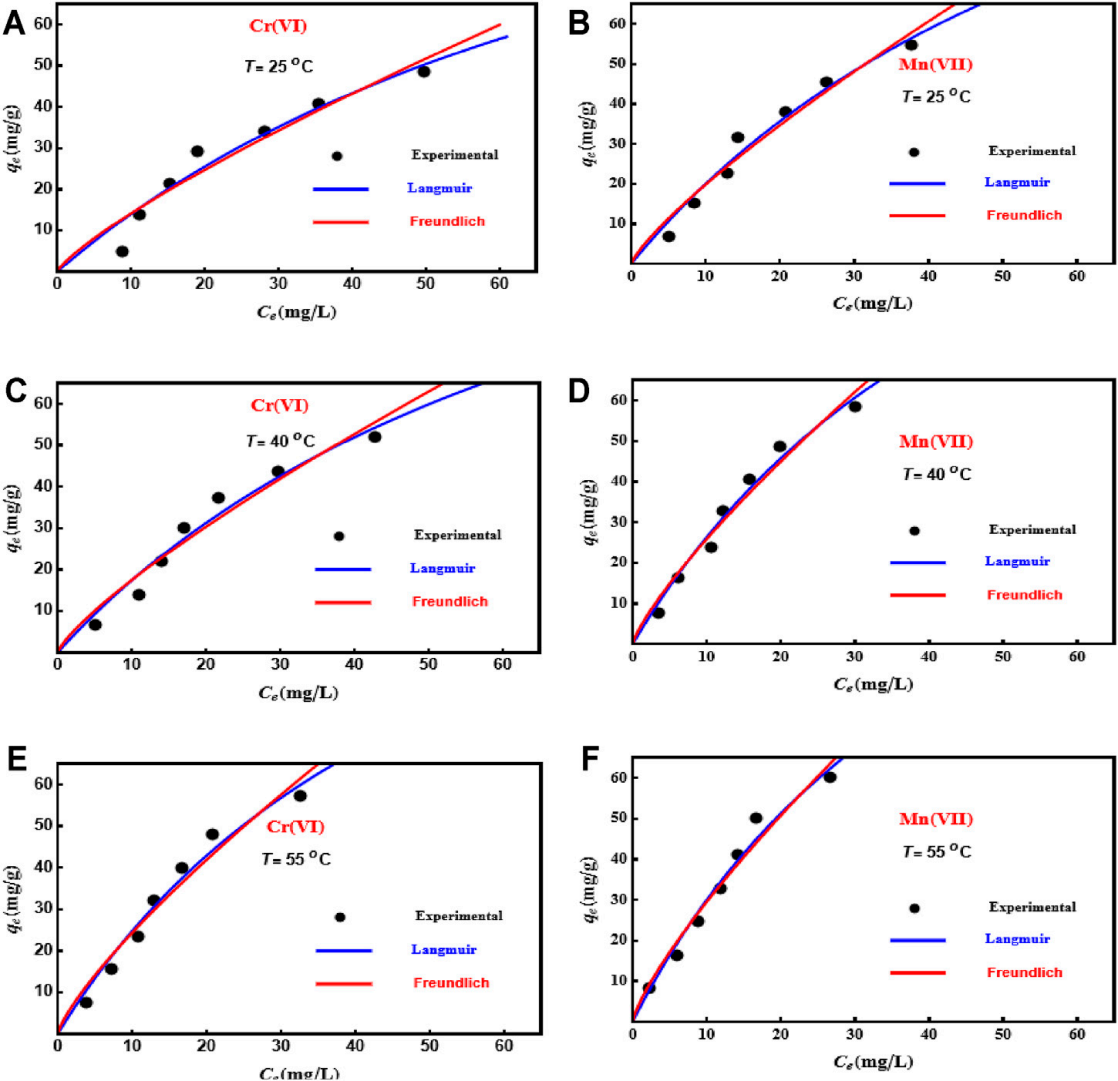
Isotherm fitting of the Langmuir and Freundlich models for both metallic ions on PD/MCM-41 composite at different temperatures.

Figure 2 displays the results of Mn(VII) and Cr(VI) adsorption isotherms on PD/MCM-41 composite including the fitting of the Langmuir and/or Freundlich equations. Table 3 gives the adjustable parameters for each classical model. Based on the $R^{2}$ values of these two models (Table 3), the adsorption data of both metal ions were well described by the Langmuir model compared to the Freundlich model at 25°C, 40°C, and 55°C. The $\chi^{2}$ values supported the application of the Langmuir model (i.e., it showed the smallest values) to analyze the interaction between the tested metal ions and the PD/MCM-41 surface; see Table 3. Consequently, Cr(VI) and Mn(VII) adsorption onto PD/MCM-41 resulted in the formation of a single layer of each tested adsorbate where it could be expected that identical functional groups were responsible for the adsorption process. The maximum Langmuir adsorption capacities (*q*_max_ $q_{max}$ ) of Cr(VI) were 144.7 (25°C), 154.8 (40°C), and 162.16 mg/g (55°C). For Mn(VI), the corresponding $q_{max}$ *q*_max_ were 166.21 (25°C), 174.39 (40°C), and 177.02 mg/g (55°C).

**TABLE 3:** Parameters of isotherm models for the adsorption of Cr(VI) and Mn(VII) on PD/MCM-41 composite

Isotherm model		*T* (°C)	Parameters		*R* ^2^	$\chi^{2}$
			*q*_max_ (mg/g)	*K*_L_ (l/mg)		
Langmuir	Cr(VI)	25	144.1	0.011	0. 9870	1.80
		40	154.3	0.013	0. 9918	3.69
		55	162.2	0.018	0.9937	2.67
	Mn(VII)	25	166.2	0.014	0. 9938	3.69
		40	174.4	0.018	0. 9953	1.95
		55	177.0	0.02	0.9949	1.52
Freundlich			*K*_F_ [(mg/g)(mg/l)^–1/n^]	1/n		
	Cr(VI)	25	2.212	0.811	0. 9836	2.01
		40	2.771	0.798	0. 9884	4.15
		55	3.992	0.785	0.9899	4.88
	Mn(VII)	25	3.087	0.807	0. 9911	8.12
		40	4.139	0.796	0. 9927	3.07
		55	4.862	0.783	0.9928	1.97

The increment of $q_{max}$ *q*_max_ at high temperature indicated that the adsorption of Cr(VI) and Mn(VII) ions on PD/MCM-41 composite was endothermic (i.e., the high temperature favored the metal–adsorbent interactions) (Seliem and Komarneni, 2016). In addition, the $q_{max}$ *q*_max_ values of Mn(VII) were higher than those of Cr(VI), thus suggesting a high selectivity of PD/MCM-41 composite for manganese ions. Respectively, *K*_F_ $K_{F}$ values from the Freundlich model also improved with temperature, thus confirming the endothermic nature of Cr(VI) and Mn(VII) adsorption. The calculated 1/n values were <1.0 (Table 3) and, therefore, PD/MCM-41 composite displayed a positive removal for both metallic ions at tested operating concentrations (Mobarak et al., 2018; Mobarak et al., 2019; Seliem and Mobarak, 2019). However, the parameters (i.e., *q*_max_ $q_{max}$ , 1/n , and *K*_F_ $K_{F}$ ) resulting from these traditional models were inadequate to define the number of ions adsorbed in each PD/MCM-41 site and the corresponding interaction mechanism (i.e., multi-docking or multi-interactions). Accordingly, the advanced SPMs were applied in this research to determine the theoretical parameters that can control the adsorbent performance and mechanism as illustrated in the next sections.

### Modeling of the Adsorption of Cr(VI) and Mn(VII) With SPMs

**FIGURE 3:**
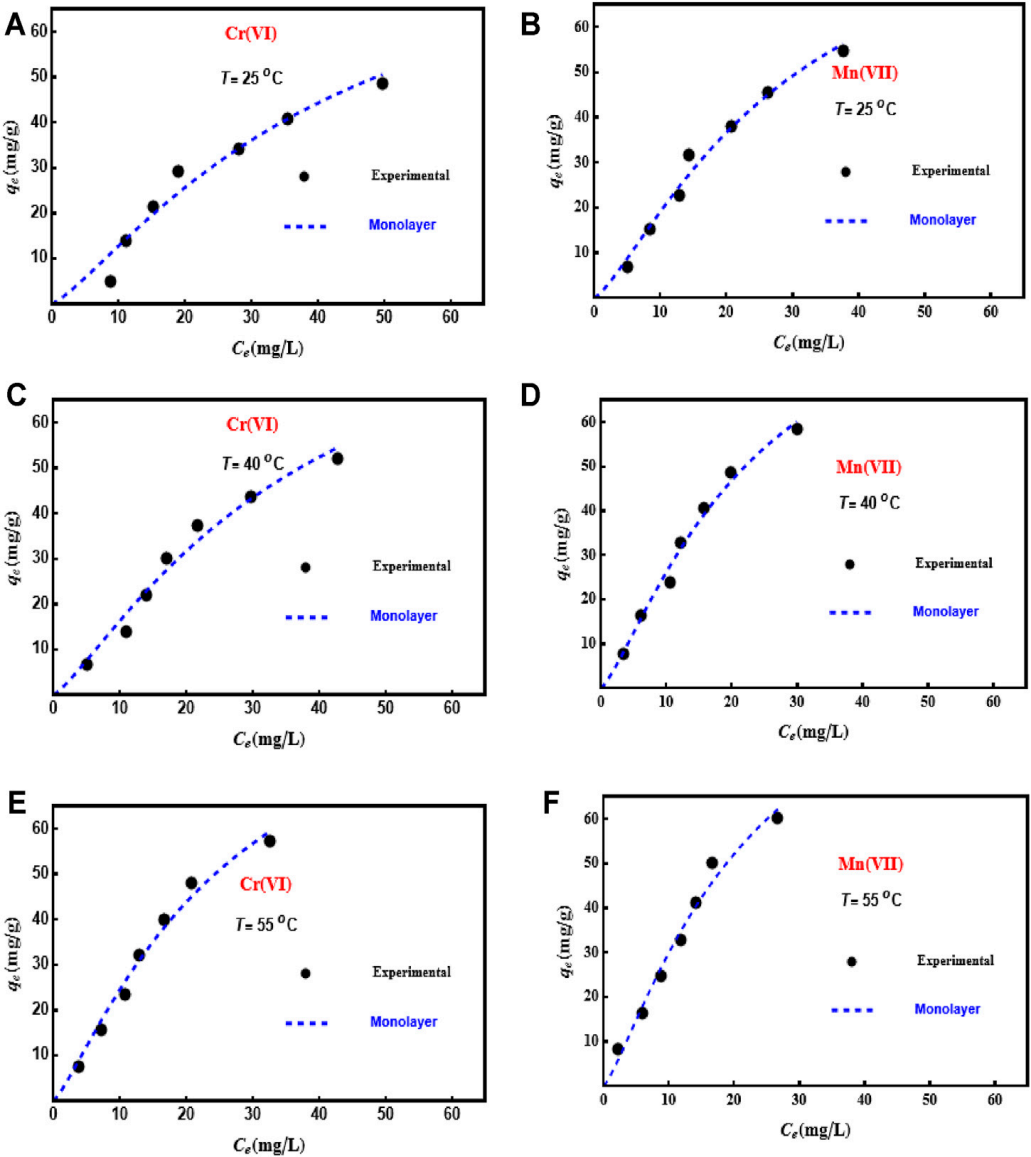
Modeling of adsorption isotherms of Cr(VI) and Mn(VII) on PD/MCM-41 composite using a monolayer statistical physics model with one adsorption energy model at 25°C–55°C.

Table 4 shows the *R* ^2^ and RMSE values where Model 1 (monolayer model with one adsorption energy) was the best to fit the experimental data (i.e., *R* ^2^ > 0.99 and RMSE values were the lowest). Therefore, the adsorption of Mn(VII) and Cr(VI) was described *via* this model, and the physicochemical (energetic and steric) parameters were interpreted according to its statistical physics-based assumptions. Figure 3 reports the fitted adsorption isotherms of Cr(VI) and Mn(VII) on PD/MCM-41 composite using this monolayer model with one adsorption site.

**TABLE 4:** Results of the modeling of Cr(VI) and Mn(VII) adsorption isotherms using different SPMs.

SPMs	Adsorbates	25°C		40°C		55°C	
		*R* ^2^	RMSE	*R* ^2^	RMSE	*R* ^2^	RMSE
Model 1	Cr(VI)	0.9926	3.09	0.9948	2.87	0.9978	3.25
	Mn(VII)	0.9945	2.58	0.9966	2.48	0.9987	2.26
Model 2	Cr(VI)	0.9603	10.39	0.9705	9.17	0.9801	9.15
	Mn(VII)	0.9713	9.39	0.9817	8.64	0.9828	9.56
Model 3	Cr(VI)	0.9506	12.09	0.9606	11.18	0.9697	10.43
	Mn(VII)	0.9584	16.15	0.994	14.17	0.9974	13.26
Model 4	Cr(VI)	0.9482	16.09	0.9507	15.06	0.9521	14.15
	Mn(VII)	0.9501	15.44	0.9436	16.11	0.9974	15.65

### Interpretation of Metal Ion Adsorption Mechanisms With Model 1

The adsorption mechanisms of both metallic ions on PD/MCM-41 were analyzed and interpreted considering the steric and energetic parameters generated by Model 1 as clarified below.

#### Interpretation of *n*, *D*_M_, and *Q*_0_ $Q_{o}\;$ Parameters

**FIGURE 4:**
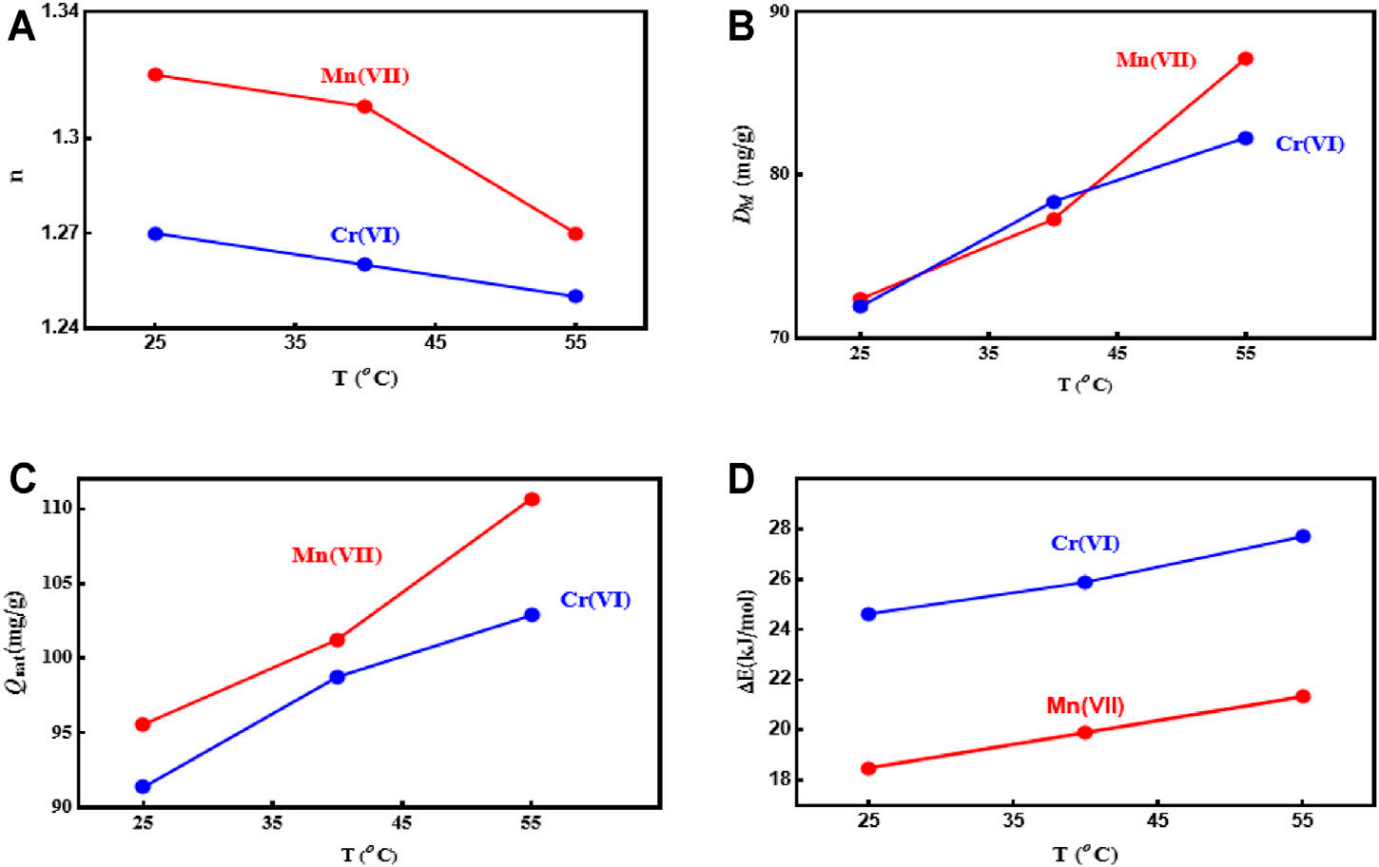
Statistical physics parameters for the adsorption of Cr(VI) and Mn(VII) on PD/MCM-41 composite at different temperatures.

The steric parameter *n* of Model 1 was used to define the number of Cr(VI) and Mn(VII) ions adsorbed per each active site of the PD/MCM-41 adsorbent. This theoretical parameter adjusted the theory of the Langmuir model where *n* is equal to unity (Li et al., 2019; Li et al., 2020; Sellaoui et al., 2017). The *n* parameter with a value above or below unity can suggest different adsorbate—adsorbent interactions (Seliem and Mobarak, 2019). A multi-docking mechanism is linked to *n* < 1 but a multi-ionic mechanism is associated to *n* > 1 (Mohamed et al., 2020; Barakat et al., 2020; Abu Sharib et al., 2021). Figure 4 shows the change in the *n* value with respect to the temperature, and the corresponding results are also given in Table 5. The *n* parameter ranged from 1.27 to 1.25 for Cr(VI) and 1.32 to 127 for Mn(VII) (i.e., *n* > 1.0 at all temperatures). So, a multi-interaction mechanism was involved in the adsorption of Cr(VI) and Mn(VII) on PD/MCM-41. For that result, it can be concluded that the active site (functional group) of the PD/MCM-41 can remove more than one ion of Cr(VI) or Mn(VII). The formation of siloxane (–Si–O–Si–) group due to the thermo-chemical activation of the diatomite was expected to be the main active site for the adsorption of both metallic ions from aqueous solutions (Mohamed et al., 2019). The indistinct decrease in the *n* value with improving the solution temperature could be related to the thermal agitation effect, which broke the interaction between the metallic ions.

**TABLE 5:** Energetic and steric parameters calculated by Model 1 for the adsorption of Cr(VI) and Mn(VII) on PD/MCM-41 composite.

	*T*	*n*	*D*_M_	*Q*_0_	Δ*E*
	(°C)		(mg/g)	(mg/g)	(kJ/mol)
	25	1.27	71.29	91.34	24.62
Cr(VI)	40	1.26	78.36	98.73	25.88
	55	1.25	82.28	102.85	27.70
	25	1.32	72.39	95.55	18.48
Mn(VII)	40	1.31	77.27	101.23	19.89
	55	1.27	87.14	110.67	21.32

Concerning the density of PD/MCM-41 active sites (*D*_M_), the temperature increment from 25°C to 55°C (Figure 4B) caused the increment of this theoretical parameter from 71.29 to 82.28 mg/g for Cr(VI) and 72.39 to 87.14 mg/g for Mn(VII). This result suggested that more siloxane sites of PD/MCM-41 were involved in the heavy metal adsorption as the solution temperature increased. Also, the interface between the siloxane adsorption sites and these metal ions at 55°C was expected to be more stable than the interaction of Cr(VI) or Mn(VII) ions in the solution. Usually, the physicochemical parameters *n* and *D*_M_ have contrasting trends as a function of adsorption temperatures (Abu Sharib et al., 2021).

The estimation of the saturation adsorption capacities of the adsorbates ( $Q_{o}$ *Q*_0_ = *n*. *D*_M_) is an essential parameter to evaluate the capability of PD/MCM-41 composite for the removal of these metal ions from solutions. *Q*_0_ $Q_{o}$ values were 91.34, 98.73, and 102.85 mg/g for Cr(VI) and 95.55, 101.23, and 110.67 mg/g for Mn(VII) at 25°C, 40°C, and 55°C, respectively; see Figure 4 and Table 5. Clearly, the *Q*_0_ $Q_{o}\;$ values increased within 25°C–55°C, and this behavior could be related to the high kinetics of Cr(VI) or Mn(VII) ions that can interact with PD/MCM-41 active sites, particularly at 55°C. The increase of $Q_{o}$ *Q*_0_ values with temperature supported the endothermic nature of the Cr(VI)/Mn(VII)–PD/MCM-41 interactions. Comparing the evolutions of *n*, *D*_M_, and *Q*_0_ $Q_{o}\;$ with the temperature, it was observed that the *Q*_0_ $Q_{o}\;$ and *D*_M_ parameters displayed an identical behavior, where *Q*_0_ $Q_{o}\;$ and *D*_M_ values increased with temperature. The *n* parameter displayed an opposite trend in comparison to *Q*_0_ $Q_{o}$ ; see Table 5. So, the *D*_M_ parameter was a key factor of the adsorption of Cr(VI) and Mn(VII) on PD/MCM-41 composite.

#### Interpretation of Adsorption Energy Parameter (Δ*E*)

The adsorption energy (Δ*E*) of the advanced monolayer Model 1 was calculated to describe the nature of the interface between Cr(VI) or Mn(VII) and PD/MCM-41 surface. The adsorption energy expression was written as given in Eq. (2) (Sellaoui et al., 2017; Dhaouadi et al., 2020a; Sellaoui et al., 2020a; Dhaouadi et al., 2020b; Sellaoui et al., 2020b; Seliem et al., 2020; Sellaoui et al., 2021b; Landin-Sandoval et al., 2021).

$$\Delta E=RT\ln\left(Cs/C_{0}\right)$$ (2)

where *R* is the universal gas constant (8.314 kJ/mol) and Cs is the solubility of Cr(VI) and Mn(VII) in water. Figure 4D and Table 5 show the Δ*E* values at three adsorption temperatures (25°C, 40°C, and 55°C). The calculated Δ*E* values were 24.62, 25.88, and 26.70 kJ/mol for Cr(VI) and 18.48, 19.89, and 21.32 kJ/mol for Mn(VII) at 25°C, 40°C, and 55°C, respectively. The adsorption energies of both adsorbates were <40 kJ/mol, suggesting that the interactions between these adsorbates and PD/MCM-41 were governed by physical forces (i.e., hydrogen bonding and van der Waals and electrostatic interactions) (Li et al., 2019; Landin-Sandoval et al., 2021; Ramadan et al., 2021).

### Interpretation of Thermodynamic Functions

In order to study the adsorption thermodynamics of these systems, different thermodynamic functions were calculated using Model 1.

#### Entropy

In order to study the adsorption entropy of the system, the homogeneity degree (order or disorder) of Mn(VII) and Cr(VI) ions on the PD/MCM-41 composite was analyzed. Adsorption entropy was obtained from the grand potential (*J*) using the total grand canonical partition function (*Z*_gc_) as follows:

$$J=-k_{B}T\;\ln Z_{\text{gc}}=-\frac{\partial \ln Z_{\text{gc}}}{\partial \beta }-T\;S_{\text{a}}$$ (3)

$$\frac{S_{\text{a}}}{k_{B}}=-\beta \frac{\partial \ln Z_{\text{gc}}}{\partial \beta }+\ln Z_{\text{gc}}$$ (4)

where *k*_B_ $\;k_{B}$ is the Boltzmann constant and *T* is the absolute temperature.

From Model 1, the entropy can be obtained by (Sellaoui et al., 2020b; Sellaoui et al., 2021b)

$$\frac{S_{\text{a}}}{k_{B}}=N_{M}\left(\ln\left[1+{\left(\frac{c}{c_{1/2}}\right)}^{n}\right]-n\;\;{\left(\frac{c}{c_{1/2}}\right)}^{n}\frac{\;\ln\left[\;\frac{c}{c_{1/2}}\right]}{1+{\left(\frac{c}{c_{1/2}}\right)}^{n}}\right)$$ (5)

**FIGURE 5:**
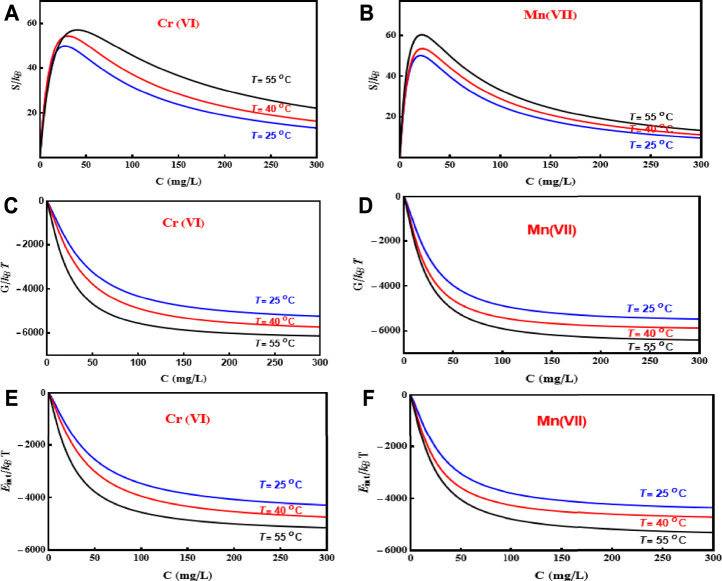
Evolution of entropy, free enthalpy, and internal energy as a function of adsorbate concentration at different temperatures for the adsorption of Cr(VI) and Mn(VII) on PD/MCM-41 composite.

The adsorption entropy of the removal processes of both metallic ions at different temperatures is shown in Figure 5 (a, b). It can be interpreted that the entropy has two states at low and high concentrations of these adsorbate ions in a solution at different temperatures. At low concentrations (before half-saturation), the entropy increased until the $c_{1/2}$ value due to the presence of a considerable number of empty active sites on PD/MCM-41 surface. Therefore, the Mn(VII) and Cr(VI) ions have a high probability to find unoccupied adsorption sites on the PD/MCM-41 composite surface. On the other hand, the adsorbate ions have a few probabilities to be attached on the PD/MCM-41 adsorbent surface at high concentration (after half-saturation) because of the adsorbent saturation. Hence, the reduction of the entropy reflected the reduced number of active sites available for heavy metal adsorption.

#### Free Enthalpy

The free enthalpy, which is related to the chemical potential ( μ ), is given by (Seliem et al., 2020)

G=μ Q (6)

According to the monolayer model with the same adsorption energy, it is rewritten as (Sellaoui et al., 2020b; Sellaoui et al., 2021b)

$$\frac{G}{k_{B}T}=n\;N_{M}\frac{\ln\left[\frac{c}{z_{\text{tr}}}\right]}{1+{\left(\frac{c}{c_{1/2}}\right)}^{n}}$$ (7)

Here, ztr=(2π m kBTh2)32 is the translation partition function, the term *h* is Planck’s constant, the term *m* is the adsorbed molecule mass, and *V* is the volume of the studied system.

At 25°C–55°C, the free enthalpy as a function of the Cr(VI) and Mn(VII) concentrations is shown in Figure 5C, D. As a result of the negative *G* values, the adsorption processes of these adsorbates were spontaneous at all temperatures. Furthermore, since the empty active sites were available on the adsorbent surface at the beginning of the adsorption process, the enthalpy started from zero and decreased with the adsorbate concentrations.

#### Internal Energy

The internal energy of adsorption was obtained by the following:

$$E_{\text{int}}=-\frac{\partial \ln Z_{\text{gc}}}{\partial \beta }+\frac{\mu }{\beta }\left(\frac{\partial \ln Z_{\text{gc}}}{\partial \mu }\right)$$ (8)

Based on the grand canonical partition function and Model 1, the internal energy becomes (Sellaoui et al., 2020b; Sellaoui et al., 2021b)

$$\frac{E_{\text{int}}}{k_{B}T}=N_{M}\frac{{\left(\frac{c}{c_{1/2}}\right)}^{n}\;\ln\left(\frac{c}{z_{tr}}\right)-n\;\ln\left(\frac{c}{c_{1/2}}\right){\left(\frac{c}{c_{1/2}}\right)}^{n}}{1+{\left(\frac{c}{c_{1/2}}\right)}^{n}}$$ (9)

Figure 5 shows the changes in internal energies versus Cr(VI) and Mn(VII) concentrations at 25°C, 40°C, and 55°C. In addition, the negative internal energy values for the two adsorbates reflected that the adsorption of Cr(VI) and Mn(VII) by PD/MCM-41 composite released energy spontaneously. Thus, the system was more stable at saturation with high adsorbate concentrations as already discussed for the free enthalpy and the internal energy; see Figure 5.

## Conclusion

Silica was extracted from the PD at 110°C/48 h and used to prepare and coat MCM-41 on the PD surface, thus producing a PD/MCM-41 composite. This composite was employed for the adsorption of metal ions, in particular Cr(VI) and Mn(VII), from solutions. The adsorption equilibrium of Cr(VI) and Mn(VII) on PD/MCM-41 was evaluated *via* experimental investigation and statistical physics analysis at three temperatures and pH 3.0. Cr(VI) and Mn(VII) adsorption was favorable and endothermic where the Langmuir model correlated satisfactorily the experimental isotherms. Statistical physics calculations indicated that a monolayer adsorption model with one adsorption site was the best to interpret the steric and energetic parameters related to the removal of these heavy metals. A multi-interaction mechanism was expected for the adsorption of Cr(VI) and Mn(VII) ions on the PD/MCM-41 surface. The adsorption capacities of Cr(VI) and Mn(VII) at saturation increased with the solution temperature, and this behavior was associated to the density of PD/MCM-41 adsorption sites. The removal of Cr(VI) and Mn(VII) using PD/MCM-41 was related to physical interactions. Entropy, free enthalpy, and internal energy suggested that the Cr(VI) and Mn(VII) adsorption processes on the PD/MCM-41 composite were spontaneous. These results revealed that the PD/MCM-41 composite can be recommended as an effective adsorbent to remove Cr(VI) and Mn(VII), and, therefore, its application could be extended for capturing other metal ions from wastewaters.

## Data Availability

The raw data supporting the conclusions of this article will be made available by the authors, without undue reservation.
